# Supplementary material for: Chromothripsis during telomere crisis is independent of NHEJ, and consistent with a replicative origin
Source: Genome Res. 2019 May;29(5):737–49. doi: 10.1101/gr.240705.118 (PMC6499312; doi:10.1101/gr.240705.118)
Supplement: Supplemental Material [file supp_gr.240705.118_Supplemental_file_1.zip › contigs/annotated_contigs/DB50/contig.2.DB50_length_481_mean_cov_5.32432432432.docx]

**DB50_length_481_mean_cov_5.32432432432**

```
AGGGCCCCCTAGATGGGGGGCGAA|GAGGGATTCTGGAACCCCTGACGGTAGTCAACGAAGGGCAGCATACAGGAAATTGGCAGTTAAC
                         >chr10:1370443-1370696 + E=1e-131 p=4e-02
AGCAGTGGAGGGTGGAAGGGATAGGACTGCAAAATGGCATTGGCCTCGAAGAAAGGCTCTTAAGGGTGGAAAAGTGGGTTTAGAAGTTG

TGTTAGGGAATTGGTAGACACCACGCCAATGTGATGAAATGAGCAGACACTACCTGAAAATCAACCACGTAACGTTCAGTTATTCCATG

CAAACCCGC|GT|GATTCTCCTGCCTCAGCCTCCCGAGTAGCTGGGATTATAGGCACCTGTCACCACGCCCAGCTAATTTTTGTTTTTT
          >chr10:2858686-2858892 + E=2e-109
TAGTAAAGATGGTAATTCAGCATGTTGGGCTGGCTGGTCTTGAACTCCTGACCTCAGGTGATCTGCCCTCCTCAGCTTCCCAAAGTGCT

GGGATTACAGGTGTGAGCTGCCACGCCCAGCCCTTTTTC
```
